# Hepcidin as a potential predictor for preoperative anemia treatment with intravenous iron—A retrospective pilot study

**DOI:** 10.1371/journal.pone.0201153

**Published:** 2018-08-08

**Authors:** Christina Wittkamp, Lisa Traeger, Ines Ellermann, Maria Eveslage, Andrea U. Steinbicker

**Affiliations:** 1 Department of Anesthesiology, Intensive Care and Pain Medicine, University Hospital Muenster, University of Muenster, Muenster, Germany; 2 Department of Pharmacy, University Hospital Muenster, University of Muenster, Muenster, Germany; 3 Institute of Biostatistics and Clinical Research, University of Muenster, Muenster, Germany; Lady Davis Institute for Medical Research, CANADA

## Abstract

Preoperative anemia occurs in about one third of patients who undergo elective surgery and is associated with an impaired outcome. Therefore, screening of preoperative anemia was established in the context of a multidisciplinary Patient Blood Management (PBM) program at the University Hospital of Muenster, Germany. Anemic patients without contraindications were treated with intravenous (IV) iron (ferric carboxymaltose) to increase their hemoglobin (Hgb) levels and hence to treat anemia prior to surgery. Interestingly, we detected a large variability in the response of Hgb levels after IV iron administration. Systemic iron homeostasis is mainly regulated by the hepatic hormone hepcidin, which regulates the cell surface expression of the sole known iron exporter ferroportin. The objective of this retrospective pilot study was to analyze the potential of hepcidin to predict the response of anemic patients to preoperative IV iron treatment measured as increase in Hgb.

Serum samples of non-anemic (n = 48), untreated anemic (n = 64) and anemic patients treated with IV iron (n = 79), in total 191 patients, were collected between October 2014 until June 2016. Serum hepcidin levels were determined and data were analyzed retrospectively.

The analysis revealed at first a correlation between serum hepcidin levels and the parameters of the iron status. Second, patients treated with IV iron showed a noticeably higher increase in their delta Hgb level between PBM consultation and surgery (0.45g/dl [0.05, 1.05] compared to patients without IV iron (0.1g/dl [-0.48, 0.73], *p = 0.03). Patients were then grouped into ‘non-responders’, defined as delta Hgb <0.6g/dl and ‘responders’, with delta Hgb ≥0.6g/dl between the day of IV iron treatment and the day of surgery. Within normal ranges and clinically unapparent, a statistically noticeable difference between responders and non-responders was found for CRP and leukocytes. Serum hepcidin levels were higher in the group of non-responders (10.6ng/ml [3.93, 34.77]) compared to responders (2.1ng/ml [0.25, 7.97], *p = 0.04).

To conclude, the data of this retrospective pilot study indicate that hepcidin might be a promising biomarker to predict a patient`s responsiveness to IV iron in preoperative anemia treatment. Prospective studies have to investigate serum hepcidin levels as a biomarker to guide physician`s decision on IV iron substitution.

## Introduction

Anemia is a public health burden that affects one quarter of the world`s population [1]. As the prevalence of anemia in patients, who undergo elective surgery, is with about 33% higher compared to the prevalence of 25% in the general population, anemia plays an important role in the preoperative management [2,3]. It is associated with a poorer outcome including a higher risk for mortality within 30 days after surgery and postoperative complications [4–6]. In addition, anemia leads to an increased perioperative use of allogenic blood transfusions (ABT), which may increase patient`s mortality [5,7,8]. IV iron administration is effective to treat preoperative anemia and can reduce the use of ABTs (reviewed by Peters *et al* in 2017) [3]. Nevertheless, not all patients respond well to IV iron in terms of an increase in Hgb levels. A broad, non-indicated IV iron infusion is dangerous, as non-utilized iron is incorporated irreversibly into the patient`s organs. Therefore, a predictor for the responsiveness of patients to increase Hgb levels after IV iron infusion is desired.

A multidisciplinary Patient Blood Management (PBM) was established in 4 German University hospitals [9]. In one of the centers, at Muenster University Hospital, an anesthesia/PBM clinic was established. Anemic patients, who were scheduled for elective surgery were screened for anemia 4 to 28 days prior to surgery and eventually treated with either 500mg or 1000mg of ferric III carboxymaltose (Ferinject^®^) [10–12]. IV iron treatment results in a higher bioavailability of iron and is able to cure iron deficiency and anemia faster than oral iron [13]. In the pre-operative setting, IV iron administration is recommended as time to surgery is generally short (within 30 days).

In the anesthesia /PBM clinic, we detected a large variability in the response of Hgb levels after IV iron administration. As a candidate biomarker to predict patient`s responsiveness to IV iron, the hepatic hormone hepcidin, the central regulator of iron homeostasis, was investigated retrospectively in the sera of 191 patients. Hepcidin is synthesized in the liver and regulated by infection and inflammation, iron availability, the erythropoetic demand, hypoxia and endocrine signals [13,14]. It regulates the expression of the sole known iron exporter ferroportin in enterocytes, macrophages and hepatocytes. Binding to ferroportin causes internalization, ubiquitination and degradation of ferroportin, which leads to decreased iron availability [15,16]. To conclude, hepcidin controls iron absorption and release, and was therefore analyzed as a potential biomarker to predict responsiveness of preoperative IV iron treatment on Hgb levels.

## Materials and methods

### Study

This study was conducted at the Department of Anesthesiology, Intensive Care and Pain Medicine of the University Hospital Muenster in Muenster, Germany from October 2014 until June 2016. The trial of PBM implementation and treatment of anemia was registered prior to patients enrollment at clinical trials.gov (NCT01820949 “Safety and Effectiveness of a Patient Blood Management (PBM) Program in Surgical Patients”, by Prof. K. Zacharowski on 03/29/2013). The Ethical Board of the Physicians Chamber of Westphalian-Lippe and the University of Muenster gave their approval (Ethical Board vote number 2013-217-B-S). The study was performed according to the Declaration of Helsinki. All patients provided a written informed consent to participate in the study, to receive IV iron supplementation (ferric carboxymaltose), if indicated, and agreed that their blood could be stored and analyzed retrospectively for serum hepcidin levels.

### Patient cohort

In this retrospective pilot study, no formal sample size calculation was performed. The participants of this study are a subgroup of the patients seen in the anesthesia/PBM clinic and were all scheduled for elective surgery [12]. Each patient underwent elective surgery for an intervention that had an increased risk of 10% or more to receive a red blood cell (RBC) transfusion in the year 2013 and were therefore seen in the anesthesia/PBM clinic between 10/2014 and 06/2016 up to one month prior to surgery [12]. 191 patients, 108 men and 83 women at the age of 22 to 87 years, were included in the study: 48 patients were non-anemic controls, 64 patients were anemic patients without IV iron treatment and 79 patients were anemic patients treated with IV iron. All patients who met the above conditions were included in the analyses.

Patients of the departments of general surgery, gynecology, neurosurgery, oral surgery, vascular and endovascular surgery, orthopedics and urology were included in the study. Patients from the department of cardiothoracic surgery were excluded, as the time frame between the visit in the anesthesia/PBM clinic and surgery was with zero to four days too short to enable IV iron to increase Hgb values prior to surgery (delta Hgb -1.3g/dl [-1.58, -0.95]).

Anemic patients presented with Hgb levels <12g/dl in women and <13g/dl in men, respectively. Patients with anemia were screened for iron deficiency. If transferrin saturation was below 20% and ferritin below 200μg/L, patients received IV iron. As published previously, anemic patients were checked for contraindications prior to an IV iron infusion. Contraindications included severe infections, hepatocellular carcinoma, liver metastases, acute severe asthma, a simultaneous oral iron medication, another IV iron preparation, iron overload, chronic renal failure and regular IV iron substitution, age ≤18 years, pregnancy, lactation and being allergic to iron [12,17]. Patients were excluded due to elevated CRP and leukocyte levels. The cutoff level for leucocytes is different in men and women (men < 10.9x10^3^/μl, women <12.68x10^3^/μl). CRP has a longer half-life than leucocytes. If leucocytes were normal, and CRP values decreasing, patients were able to receive IV iron. After the exclusion of contraindications, anemic patients received either 500mg or, in three cases, 1000mg of ferric III carboxymaltose (Ferinject^®^, Vifor Pharma, St. Gallen, Switzerland).

### Patients’ data

Blood samples were analyzed in the Center for Laboratory Medicine of the University Hospital Muenster. The blood parameters Hgb, hematocrit, transferrin protein, ferritin, serum iron, transferrin saturation, CRP, leukocytes, erythrocytes, mean corpuscular volume (MCV), mean corpuscular hemoglobin (MCH), mean corpuscular hemoglobin concentration (MCHC) and thrombocytes were analyzed retrospectively by screening patients’ electronic data files. All blood parameters were measured at baseline blood draw. Only Hgb levels were measured twice (at baseline and directly prior to surgery). Complementary patients’ data included gender, date of birth, current age at the visit in the anesthesia/PBM clinic, corresponding surgical department, surgery and date of surgery, date of baseline blood draw as well as days between the baseline blood draw and the surgery were collected in an excel file for the analysis.

### Serum hepcidin levels

Patients’ serum samples were stored at -80°C until further use. Hepcidin serum levels were measured with the hepcidin 25 (bioactive) enzyme-linked immunosorbent assay (ELISA) EIA-5782 (DRG^®^ Instruments GmbH, Marburg, Germany) on a 96-well plate according to the manufacturer`s instructions. Measurements were performed in duplicates on a BioTek photometer (EL808, BIOTEK® Instruments INC., Winooski, VT, USA) equipped with the Gen5 software (Gen5 Data Analysis Software, BIOTEK® Instruments INC.).

### Delta Hgb

Delta Hgb levels were calculated as the difference between the Hgb level at the time of PBM consultation and the Hgb level prior to surgery. From the clinical perspective, in the IV iron treatment group patients with a delta Hgb ≥0.6g/dl were defined as ‘responders’ and patients with a delta Hgb <0.6g/dl as ‘non-responders’. Hepcidin, CRP, leukocytes, age, the number of days between IV iron and surgery, the iron status and erythrocyte parameters were compared between responders and non-responders. For the subanalysis 40 anemic patients treated with IV iron were grouped into responders and non-responders. The delta Hgb could only be calculated, if the following criteria were met:

- Second blood draw prior to surgery. Patients without a measurement of Hgb in the second blood draw could not be included in the analysis of delta Hgb.
- A minimum time span of 4 days between IV iron infusion and surgery.
- A maximum time span of 28 days between IV iron infusion and surgery.
- No preoperative administration of red blood cell concentrates.

### Statistical analysis

All statistical analyses and graphical creations were performed with GraphPad Prism 6^®^ (GraphPad Software Inc., La Jolla, California) and Microsoft^®^ Excel 2013. The results are expressed as median [Q1, Q3]. Spearman’s rank correlation coefficient r_s_ with a two-tailed p-value was used for correlation analysis. For the comparison of Hgb values between non-anemic, anemic and anemic patients treated with IV iron Kruskal-Wallis test for multiple comparisons was used. For pairwise comparisons, Mann-Whitney U-Test with a two-tailed p-value was used. In addition, multivariable linear regression analyses were performed in order to adjust for potential confounders. Delta Hgb in anemic patients was modeled dependent on IV iron treatment, the number of days between PBM visit and surgery, age, and gender. In the analysis of delta Hgb in anemic patients treated with IV iron, the serum hepcidin levels, the number of days between PBM visit and surgery, age, and gender were included.

Different cut-offs of the delta Hgb with a correction for multiple testing were tested using the Wilcoxon Mann-Whitney-U test. These analyses were performed using the maxstat package for R (version 3.4.3) using pmethod = “Lau94” for determination of the upper bound of the p-value [18]. CRP concentrations <0.5mg/dl, the lowest value provided by the central laboratory interpreted as normal, was set to 0.4mg/dl to perform calculations.

All inferential statistics are intended to be exploratory (hypothesis generating), not confirmatory, and are interpreted accordingly. No adjustment for multiple testing was applied. P-values p ≤ 0.05 are considered statistically noticeable.

## Results

### Hepcidin correlated with the iron status

**Fig 1:**
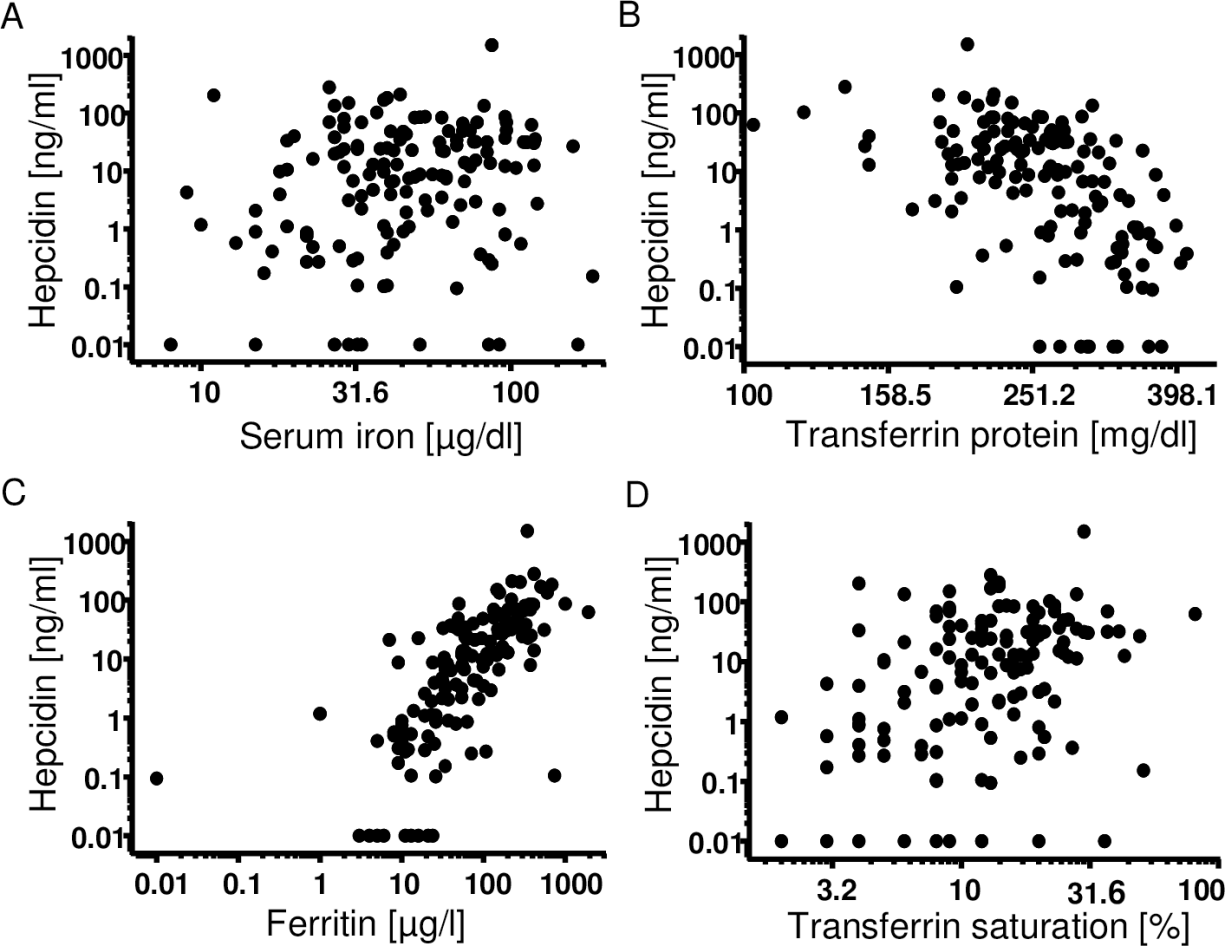
Correlation of serum hepcidin levels with parameters of the iron status. Serum hepcidin levels of the three patient groups of non-anemic controls, anemic patients treated with IV iron and anemic patients without IV iron treatment, were correlated to the iron parameters. The x- and y-scales are depicted logarithmic (log10). Values of “0” were replaced with 0.01 in order to depict them in the logarithmic graph. Serum hepcidin levels correlated with **(A)** serum iron levels (n = 146 XY-pairs (n = 9 non-anemics, n = 58 anemics without IV iron, n = 79 anemics plus IV iron); r = 0.19; Spearman *p = 0,02), **(B)** transferrin protein levels (n = 143 XY-pairs (n = 6 non-anemics, n = 58 anemics without IV iron, n = 79 anemics plus IV iron); r = -0,55; Spearman *p<0.0001), **(C)** ferritin levels (n = 143 XY-pairs (n = 6 non-anemics, n = 58 anemics without IV iron, n = 79 anemics plus IV iron); r = 0,75 Spearman *p<0.0001) and **(D)** transferrin saturation (n = 142 XY-pairs (n = 5 non-anemics, n = 58 anemics without IV iron, n = 79 anemics plus IV iron); r = 0.37; Spearman *p<0.0001).

As hepcidin is the iron regulatory protein and was investigated as a potential biomarker, the correlation with parameters of the iron status was analyzed. Nowadays, measurement of the iron status is required prior to the decision on IV iron treatment. Hepcidin has a skewed distribution. Therefore, Spearman’s rank correlation coefficient was used. Serum hepcidin levels correlated with serum iron levels (Fig 1A, r = 0.19, Spearman *p = 0.02, n = 146), transferrin protein (Fig 1B, r = -0.55, Spearman *p<0.0001, n = 143), ferritin levels (Fig 1C, r = 0.75, Spearman *p<0.0001, n = 143), and transferrin saturation (Fig 1D, r = 0.37, Spearman *p<0.0001, n = 142). The correlation coefficient was highest between ferritin and hepcidin. The data indicate that serum hepcidin levels correlated well with the iron status.

### Preoperative treatment with IV iron increased Hgb levels prior to elective surgery

**Fig 2:**
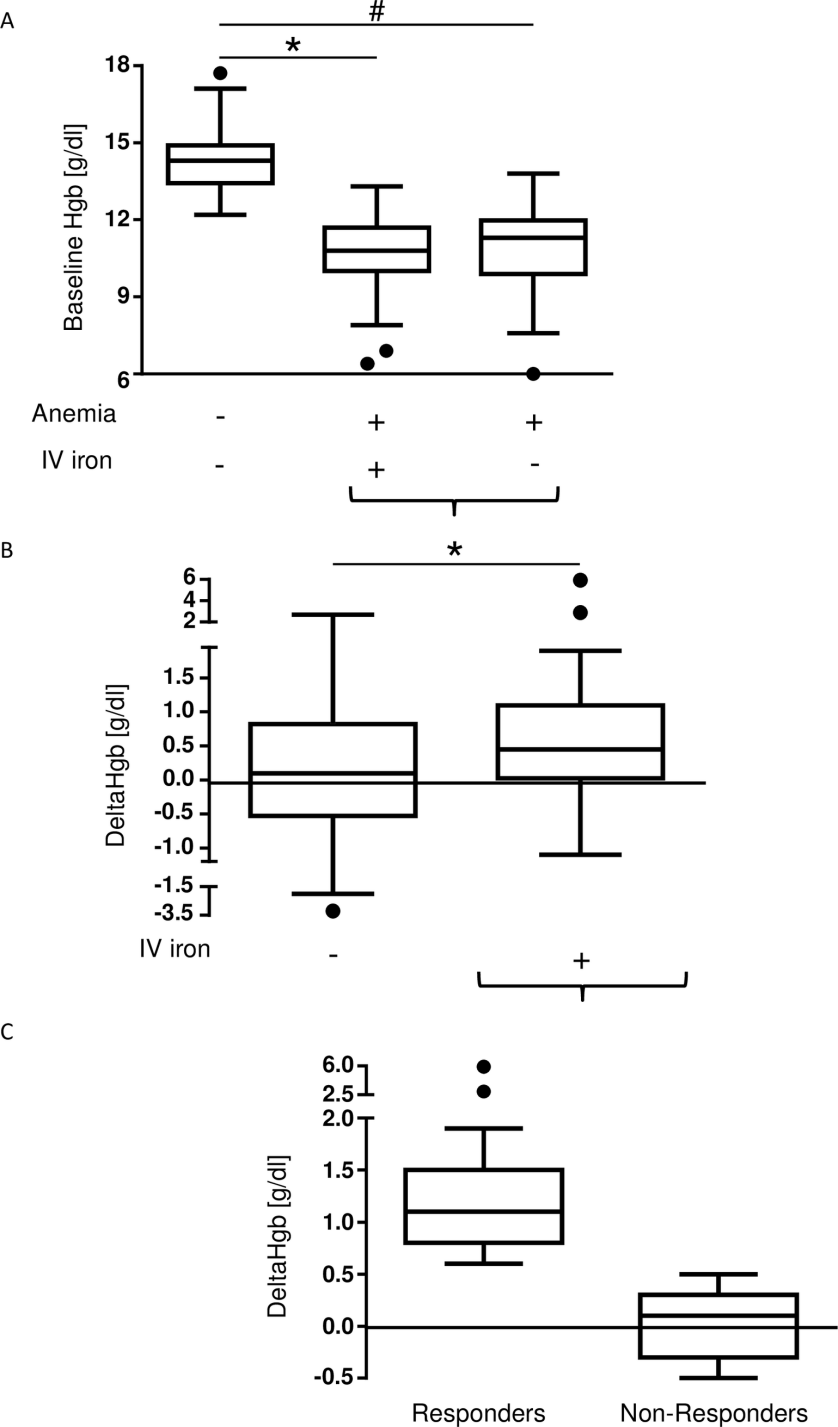
Delta Hgb is higher in anemic patients who received IV iron. Delta Hgb values represent the difference of the hemoglobin value that was measured directly prior to surgery and the baseline hemoglobin level. **(A)** Baseline Hgb values were higher in non-anemic patients (14.3g/dl [13.48, 14.9], n = 48) than in both anemic patients with (10.8g/dl [10.05, 11.7], n = 79, *p<0.0001) and without (11.3g/dl [10.1, 11.93], n = 64, ^#^p<0.0001) IV iron treatment. **(B)** Delta Hgb values were higher in anemic patients who were administered with IV iron (n = 40) (0.45g/dl [0.05, 1.05] vs. 0.1g/dl [-0.48, 0.73], *p = 0.03) compared to anemic patients without IV iron treatment (n = 26). **(C)** Patients with an increase in Hgb levels of ≥0.6g/dl were considered responders. Delta Hgb values were higher in the group of responders (1.1g/dl [0.8, 1.45], n = 19 patients) compared to the delta Hgb values in the group of anemic non-responders (0.1g/dl [-0.3, 0.3], n = 21 patients).

Of the 191 patients recruited for this retrospective study, Hgb levels were determined 4–30 days prior to elective surgery and directly prior to surgery. Fig 2A depicts baseline Hgb levels of non-anemic, anemic plus IV iron and anemic patients without IV iron treatment. Baseline Hgb levels were 14.3g/dl [13.48, 14.9] (non-anemics, n = 48), 10.8g/dl [10.05, 11.7] (anemics plus IV iron, n = 79), and 11.3g/dl [10.1, 11.92] (anemics without IV iron, n = 64), respectively. Anemic patients who had received IV iron (n = 40) in the PBM/anesthesia clinic, had higher delta Hgb levels (0.45g/dl [0.05, 1.05]) compared to untreated anemic patients (n = 26) (0.1g/dl [-0.48, 0.73]; *p = 0.03) (Fig 2B). The data indicate that preoperative IV iron administration increased Hgb levels in the preoperative setting in the current cohort of patients planned for elective surgery, comparable to the data published for the whole patient cohort [12].

Interestingly, delta Hgb values of anemic patients treated with IV iron revealed an inter-individual variability in the responsiveness to IV iron with a range from -1.1g/dl to 5.9g/dl.

‘Responders’ were defined as delta Hgb ≥0.6g/dl and an Hgb <0.6g/dl as ‘non-responders’. Responders (n = 19) presented a delta Hgb of 1.1g/dl [0.8, 1.45], which was obviously higher compared to non-responders (n = 21, delta Hgb 0.1g/dl [-0.3, 0.3]) (Fig 2C).

The results are supported by a multivariable regression analysis performed for the potential confounding effects of 1.) number of days between visit and receipt of IV iron and surgery, 2.) age of the patient and 3.) gender (Table 1). The effect of IV iron is statistically noticeable (Hgb -0,6g/dl, *p = 0.03).

**Table 1:** Multivariable regression analysis for potential confounding effects.

Parameter	Regression coefficient	95% confidence interval	p-value
Intercept	0.028	[-1.759, 1.815]	0.976
Anemia with IV iron	-0.595	[-1.144, -0.047]	0.033
Anemia without IV iron	Reference	-	-
Number of days	0.026	[0.008, 0.044]	0.006
Gender (female)	0.28	[-0.291, 0.85]	0.337
Gender (male)	Reference	-	-
Age [years]	0.002	[-0.023, 0.026]	0.901

Table 1 shows the results of a multivariable regression analysis, in which the effect of IV iron on Hgb levels has been analyzed adjusted for the potential confounding factors a) number of days, b) age of the patients and c) gender.

### Patients‘ characteristics of responders and non-responders did not differ

In order to elucidate the reason for the unresponsiveness to IV iron, patients of both groups were characterized (Table 2). There was no statistically noticeable difference between the groups in terms of gender and age (responders: 69 years [59, 76], vs. non-responders: 73 years [64, 78]). The number of days between IV iron receipt and surgery was smaller in non-responders compared to responders (responders: 18 days [6, 27] vs. non-responders: 8 days [6, 14]). The iron status including serum iron, transferrin protein, transferrin saturation and ferritin as well as the erythrocyte parameters MCV, MCH and MCHC were also analyzed.

**Table 2:** Characteristics of responders and non-responders.

	Responders		Non-Responders		
Parameter	Number of patients (n)	Median [Q1, Q3]	Number of patients (n)	Median [Q1, Q3]	P-value
Age [years]	19	69 [59, 76]	21	73 [64, 78]	0.37
Days between IV iron and surgery	19	18 [6, 27]	21	8 [6, 14]	0.2
Serum iron [μg/dl]	19	47 [19, 56.5]	21	36 [30, 45]	0.41
Transferrin protein [mg/dl]	19	293 [230, 320.5]	21	258 [222, 290]	0.31
Transferrin saturation [%]	19	14 [5.5, 16]	21	11 [7, 14]	0.74
Serum ferritin [μg/l]	19	36 [15, 54.5]	21	54 [33, 146]	0.07
MCV [fl]	19	85.2 [73.4, 90.4]	21	86.5 [79.7, 90.2]	0.63
MCH [pg]	19	28 [22.05, 30.25]	21	26.9 [25.8, 28.9]	0.91
MCHC [g/dl]	19	31.7 [30.25, 33.35]	21	31.8 [31, 32.6]	0.85

Table 2 shows the parameters age and the iron parameters serum iron, transferrin protein, transferrin saturation and serum ferritin levels as characteristics of responders and non-responders, all measured in the baseline blood draw at the time of PBM consultation. Table 2 shows the number of investigated patients and the respective median and IQR for each parameter. A statistically noticeable difference in the comparison of responders and non-responders was not observed.

Serum iron levels (responders: 47μg/dl [19, 56.5], vs. non-responders: 36μg/dl [30, 45]), transferrin protein (responders: 293mg/dl [230, 320.5], vs. non-responders: 258mg/dl [222, 290]) and transferrin saturation (responders: 14% [5.5, 16], vs. non-responders: 11% [7, 14]) were within similar ranges in both groups of responders and non-responders. The same applies for the erythrocyte parameters MCV (responders: 85.2fl [73.4, 90.4], vs. non-responders: 86.5fl [79.7, 90.2]), MCH (responders: 28pg [22.05, 30.25], vs. non-responders: 26.9pg [25.8, 28.9]) and MCHC (responders: 31.7g/dl [30.25, 33.35], vs. non-responders: 31.8g/dl [31, 32.6]).

Non-responders showed higher ferritin levels than responders. The p-value was rather small, so that relevant differences can be expected (responders: 36μg/l [15, 54.5], vs. non-responders: 54μg/l [33, 146], p = 0.07).

The data show that there was no difference in patients’ characteristics in responders compared to non-responders that could have guided physicians in the decision on treatment or predict how a patient`s responsiveness to IV iron might be. Therefore, additional parameters had to be screened.

### The inflammatory parameters leukocytes and CRP of responders and non-responders differed

**Fig 3:**
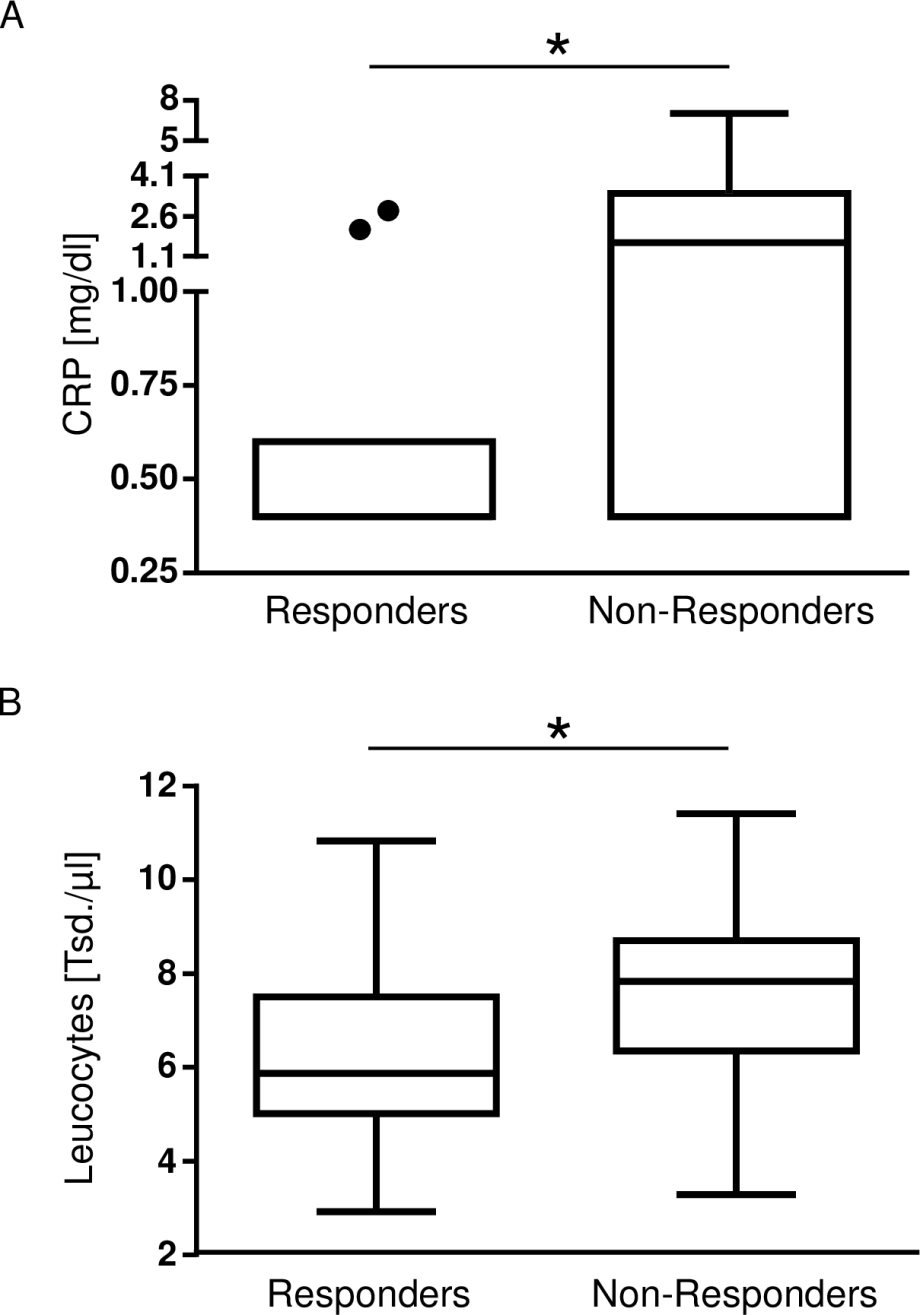
Comparison of the inflammatory parameters leukocytes and CRP in the groups of responders and non-responders. CRP levels and leukocytes as parameters of inflammation were compared between the groups of responders and non-responders. **(A)** CRP levels were higher in the group of non-responders (responders: 0.4mg/dl [0.4, 0.5], n = 11, vs. non-responders: 1.6mg/dl [0.4, 2.5], n = 13, *p = 0.04). **(B)** Leucocytes were higher in the group of non-responders, too (responders: 5.87 *10^3^/μl [5.2, 7.1], n = 19, vs. non-responders: 7.84 *10^3^/μl [6.39, 8.6], n = 21, *p = 0.04).

It is important to preclude an acute infection prior to the IV iron administration, because pathogens themselves require iron for replication and growth. The effects of IV iron administration in states of acute or chronic infection are not yet completely known [19,20]. CRP and leukocytes are standard laboratory parameters for inflammation and available in patients prior to elective surgery. The comparison of CRP levels (0.4mg/dl [0.4, 0.5], n = 11, vs. 1.6mg/dl [0.4, 2.5], n = 13, *p = 0.04, Fig 3A) and leucocytes (5.87Tsd/μl [5.2, 7.1], n = 19, vs. 7.84Tsd/μl [6.39, 8.6], n = 21, *p = 0.04, Fig 3B) between responders and non-responders showed that CRP values and leukocyte numbers were different within the groups of responders and non-responders although clinically still in a low range. Nevertheless, as even subclinical inflammation is a frequent driver of increased hepcidin expression and functional iron deficiency, inflammation can never be completely excluded. Leucocytes were within normal ranges and CRP values normal to slightly induced. These results implicate that serum CRP and leukocyte levels might have to be carefully considered to predict the responsiveness to preoperative IV iron treatment in anemic patients. With regard to the reduced number of patients in the evaluation of CRP levels this result has to be interpreted with high caution.

Serum hepcidin levels correlated statistically noticeable with CRP levels (n = 131 XY-pairs (n = 33 non-anemics, n = 47 anemics without IV iron, n = 51 anemics plus IV iron); r = 0.21; Spearman *p = 0.01), but not with leukocyte levels.

### Hepcidin may serve as a potential biomarker to predict the responsiveness to IV iron

**Fig 4:**
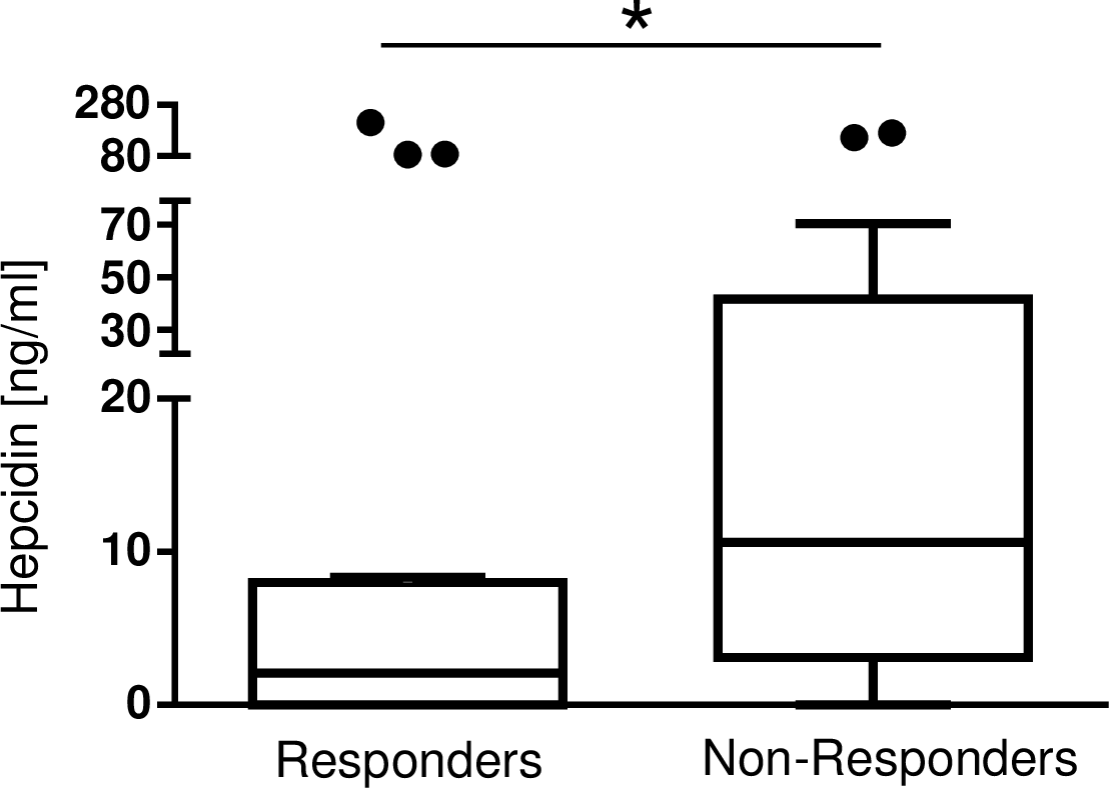
Comparison of hepcidin levels in the groups of responders and non-responders. Serum hepcidin levels were lower in anemic patients with a good response to IV iron (2.07ng/ml [0.25, 7.97], n = 19) compared to anemic patients with an impaired response (10.62ng/ml [3.93, 34.77], n = 21; *p = 0.04).

An increase in infection parameters was a contraindication for IV iron administration. It is conceivable that an inflammatory reaction had started or was ongoing, but could not be detected by standard laboratory parameters. As hepcidin is induced by inflammation, it might be responsible for the unresponsiveness to IV iron treatment. Serum hepcidin levels were lower in responders (2.07ng/ml [0.25, 7.97], n = 19) than in non-responders (10.62ng/ml [3.93, 34.77], n = 21, *p = 0.04) (Fig 4). Due to clinical relevance, the range of delta Hgb of 0.4–1.2g/dl was tested with a similar number of patients (n) in both groups. The optimal cut-off is 0.5g/dl. Using this cut-off of 0.5g/dl, serum hepcidin levels are statistically noticeable (*p = 0.04), the multiplicity adjusted p-value is p = 0.158. If the group sizes can differ in the number of patients, the optimal cutoff is a delta Hgb of 1.3g/dl (n = 6, vs n = 34). Testing the difference in serum iron levels in these two groups, the statistical significance remains (without adjustment for multiple testing: *p = 0.004, adjusted for multiple testing: *p = 0.046). As the current study is a retrospective study to generate hypotheses, the cut-off for the responsiveness in delta Hgb has to be determined in a prospective study- of note taking the clinical relevance of the delta Hgb into account.

In addition, a multivariable regression analysis of delta Hgb adjusting for the potentially confounding effects in the subcohort of patients with IV iron treatment was performed. Besides hepcidin the potentially confounding effects of 1.) number of days between visit and receipt of IV iron and surgery, 2.) age of the patient and 3.) gender were included (Table 3). This analysis supports the trend of lower delta Hgb at high serum hepcidin levels.

**Table 3:** Multivariable regression analysis of delta Hgb.

Parameter	Regression coefficient	95% confidence interval	p-value
Intercept	-0.14	[-2.098, 1.818]	0.885
log(Hepcidin+1)	-0.099	[-0.321, 0.124]	0.374
Number of days	0.03	[0.009, 0.05]	0.007
Gender (male)	-0.34	[-1.081, 0.401]	0.358
Gender (female)	Reference	-	-
Age [years]	0.011	[-0.018, 0.039]	0.456

Table 3 shows the results of a multivariable regression analysis of delta Hgb adjusted for the potential confounding factors hepcidin, number of days between receipt of IV iron and surgery, age of the patient and gender.

## Discussion

Perioperative anemia treatment is challenging, as not all patients respond well to IV iron. A biomarker to predict the responsiveness of anemic patients to IV iron would be helpful for clinical use. The current pilot study aimed to investigate whether serum hepcidin levels may predict the increase in Hgb levels after IV iron administration in anemic patients. The results indicated that 1.) delta Hgb was higher in patients, who received IV iron compared to untreated patients, 2.) among the patients treated with IV iron different responses (measured as an increase in Hgb levels) to the iron administration in the sense of responders and non-responders were observed, 3.) serum hepcidin levels correlated well with the iron status of a patient and might be a useful biomarker in the preoperative setting to treat anemia, and last but not least, 4.) serum hepcidin differed between responders and non-responders and might be a useful biomarker to predict a patient’s responsiveness to IV iron.

Of note, we want to have our study interpreted with high caution, as the small sample size of the study is a limitation. As described in the publication about the anesthesia/PBM clinic, the patient cohort of a University Hospital differs in a) the individual time span between presentation in the anesthesia/PBM clinic, b) in each patient’s morbidities and co-morbidities and c) surgical intervention and an individual perioperative course. A multicenter, prospective trial with a higher sample size is required to evaluate the use of serum hepcidin as a biomarker for the responsiveness of preoperative IV iron in anemic patients.

A categorization of anemia into iron deficiency anemia (IDA), anemia of chronic disease (ACD) or a combination of both (IDA+ACD) was performed. In the responders, more patients presented with IDA than in the non-responder group. In contrast, non-responders had more often a combination of IDA and ACD than responders. Of note, patients with a combined anemia (IDA+ACD) may demonstrate low serum hepcidin levels despite systemic inflammation or infection [21]. Therefore, future studies have to investigate, if IV iron administration is safe in patients suffering from combined anemia (IDA+ACD).

One obvious limitation of the current pilot study is the time span between IV iron substitution and surgery. Due to clinical reasons- and the implementation of a clinical relevant anemia clinic, we had to adjust the time span according to the surgical needs. Therefore, patients presented for anemia screening and treatment between 4–28 days prior to surgery. In a future prospective trial, the time frame has to be set similarly for patients included (i.e. 7 days).

To have a biomarker that predicts a patient’s benefit from an IV iron administration is not only relevant in the preoperative setting but also in other settings that require IV iron treatment. Wegmüller *et al*. measured serum hepcidin in young anemic children in Gambia to decide on IV iron treatment. In this setting, it is also very important to have a screening parameter to avoid unessential iron application [22]. Additionally, Nielsen *et al*. suggest the importance of a goldstandard biomarker that can be used to prevent insufficient and excessive iron administration [23]. It is important to investigate the cause of failure of IV iron therapy [23]. High serum hepcidin concentrations might be the reason why patients did not respond to the IV iron administration.

If hepcidin is upregulated, it internalizes and degrades ferroportin. As a result, intestinal iron absorption is reduced and iron is trapped within the iron stores in macrophages and hepatocytes [15,24]. At the same time, externally substituted iron is not absorbed when delivered orally. *Bregman et al*. reported that high levels of serum hepcidin correlated with non-responsiveness to orally administered iron in patients with iron deficiency anemia [25]. In states of hepcidin induction, IV iron can be incorporated into the iron storage sites, but not be released from the latter. *Beutler* supposed that in cases of high dose IV iron substitution, IV administered iron that is not used for erythropoiesis directly, ends up as storage iron and is not available for erythropoiesis [26]. *Nemeth et al*. reported that in anemia of inflammation, erythropoiesis is suppressed by direct cytokine interactions with the bone marrow [27]. In addition, cytokines interact with renal cells and suppress the synthesis of erythropoietin [27]. In anemia of cancer, erythropoietin levels were reported to be inadequate low [28]. As anemia of cancer goes along with inflammation and thus elevated hepcidin levels, this provides another reason for failure of IV iron therapy under elevated serum hepcidin levels.

In contrast to other studies that investigated possible predictors for the increase of serum Hgb levels after iron administration (for example *Drakou et al*.), our definition of the ‘response-group’ is not a deltaHgb value of 1g/dl or even more. Clinically, an increase of ≥ 0.6g/dl is relevant, because most of our patients received 500mg of FCM and not 1g of FCM. The time span between iron administration and second blood draw was for some patients only 4 days (in contrast to *Drakou et al*., who determined the second Hgb value one month after IV iron treatment). These two facts, the dose of 500mg of FCM and in some cases the short period of time between IV iron administrations must be respected for the delta Hgb cut off value, led to a delta Hgb cut off value of 0.6g/dl as appropriate. In addition, testing different delta Hgb levels with a correction for multiple testing using the standardized Wilcoxon Mann-Whitney-U test [18] led to similar results.

As the current study is a retrospective study to generate hypotheses, the cut-off for the responsiveness in delta Hgb has to be determined in a prospective study- of note taking the clinical relevance of the delta Hgb into account.

According to current guidelines, transferrin saturation and ferritin are required parameters to decide on IV iron treatment. Theoretically, ferritin might also serve as a possible predictor to detect early inflammation or full iron stores, as it is not only the iron storage protein, but also an acute phase protein. In iron deficiency, serum ferritin generally correlates with the iron stores of the body. However, in cases of inflammation, chronic liver diseases and malignant processes ferritin is increased independently of the body’s iron status by acute phase processes [29]. A consensus statement also indicated that IV iron substitution is reasonable in patients with anemia of chronic disease including cancer or chronic inflammatory diseases such as cardiac heart failure, inflammatory bowel syndrome or chronic kidney disease, if ferritin is below 300ng/ml and the transferrin saturation is below 20% [30]. A serum ferritin of 300μg/L as recommended by Cappelini *et al*. is highly controversial. Ferritin between 200–300μg/L and a TSAT below 20% do not exclude an ACD. An evaluation with additional parameters such as sTFR or sTFRF index may help to distinguish between ACD and ACD+IDA in these patients and is warranted prior IV iron treatment.

Acute inflammation leads to an increase in CRP values as an acute phase protein and leukocyte levels. In contrast to this, *in vivo* hepcidin seems to be more influenced by iron deficiency than by inflammation [21,31]. Theurl *et al*. suggested that regulatory hepcidin pathways that are induced by iron deficiency are able to convert the stimulation of hepcidin expression that is induced by inflammation [21]. This implies that hepcidin, in contrast to exclusively inflammatory parameters such as CRP and leukocytes, might be a useful parameter for diagnosis of a true iron deficiency in patients with anemia and active inflammation. Non-responsiveness is most conceivably caused by an induction of the inflammatory parameters. Interestingly, four patients of the current cohort, were severely iron depleted with ferritin levels below 30μg/l, but still belonged to the non-responder group. Serum hepcidin levels were higher in these patients and therefore a potential reason for the non-responsiveness.

Other investigators have also analyzed the potential of hepcidin as a biomarker in other settings than in preoperative anemia treatment: Tables 4 and 5 show six studies that investigated the role of hepcidin as a potential biomarker for the response to iron treatment and/or a correlation between serum levels of hepcidin and ferritin [32–37].

**Table 4:** Four studies that analyzed hepcidin as a potential biomarker and/or the correlation between hepcidin and ferritin.

Author	Year	Journal	Setting	Biomarker	Hepcidin / ferritin
Drakou[32]	2016	Blood Cells Mol Dis.	78 predialysis CKD (stage III-IV) patients with anemia and ferritin levels < 100ng/ml, treatment with 1g of FCM, classification as responders and non-responders (one month after FCM administration), investigation of hepcidin levels in both groups	+	+
Steensma[33]	2015	Blood	Cancer patients with chemotherapy-associated anemia, treatment with darbepoetin alfa and IV iron, classification as responders and non-responders, investigation of hepcidin levels in both groups	+	**-**
Sonnweber[34]	2011	Nephrol Dial Transplant.	24 haemodialysis patients, treatment with a single dose of 100mg IV iron sucrose (intervention group) or with saline (control group), blood draws before iron administration at day 0, after 48h and 1 week, immediately before dialysis, measurement of serum hepcidin retrospectively	+	+
Weiss[35]	2009	Eur J Clin Invest.	20 chronic haemodialysis patients, baseline measurement of hepcidin levels, treatment with (a) erythropoietin, (b) IV iron (iron saccharose) and (c) a combination of iron and erythropoietin, investigation if dialysis procedure influences hepcidin levels and how treatment (erythopoietin and iron) change hepcidin levels	/	+

Table 4 depicts four studies, that also investigated the potential of hepcidin as a biomarker for the response to iron treatment and/or the correlation between hepcidin and ferritin. Three studies were performed in CKD patients, one in patients under cancer therapy. Three authors are in accordance (+) with the current pilot study and concluded that hepcidin levels might predict the response to iron treatment (column “biomarker”). One study did not investigate (/) the role of hepcidin as a potential biomarker. Three of the studies listed in Table 4 stated that there was a statistically noticeable correlation between hepcidin and ferrtitin (+); one did not show a correlation between these two parameters (-) (last column “hepcidin/ferritin”).

**Table 5:** Two studies that analyzed hepcidin as a potential biomarker and/or the correlation between hepcidin and ferritin.

Author	Year	Journal	Setting	Biomarker	Hepcidin / ferritin
Tessitore[36]	2010	Nephrol Dial Transplant.	56 hemodialysis patients, treatment with 1g of IV iron (ferric gluconate), measurement of hepcidin to predict haemoglobin increase after iron treatment and the correlation with markers of the iron status, inflammation and erythropoietic activity, distinction between responders (>1g/dl) and non-responders according Hgb-increase after iron treatment	-	+
Gaillard[37]	2016	PLoS One.	626 patients with non-dialysis CKD and iron deficiency, randomized to IV iron (ferric carboxymaltose) targeting higher or lower ferritin or oral iron, hepcidin measurement in 61 patients. Initial dose of iron was administered on day 0: 1000mg FCM (high ferritin group) and 200mg FCM (low ferritin group) if ferritin was <100μg/l, administration of FCM every four weeks during weeks 4–48 FCM at a dose of 500mg or 1000mg FCM (high ferritin group) depending on the ferritin level and in the low at a dose of 200mg iron (low ferritin group) if ferritin was <100μg/l. Oral iron therapy: ferrous sulfate (100mg iron) twice daily, analysis of correlations between hepcidin and ferritin within each treatment group at weeks 8 and 24, Hgb increase also measured in week 8 and week 24.	-	+

Table 5 lists two studies, that also investigated the potential of hepcidin as a biomarker for the response to iron treatment and/or the correlation between hepcidin and ferritin. Both studies were performed in CKD patients. Both publications did not support hepcidin as a biomarker (-), but indicated a statistically noticeable correlation between hepcidin and ferritin (+).

Iqbal *et al*. criticized the use of hepcidin as a biomarker for the responsiveness to IV iron therapy, because hepcidin immunoassays can detect the inactive form of hepcidin as well as the biological active variant (hepcidin-25) [38]. In addition, the measurement results could vary tenfold using different assays.–This fact implicates a difficulty in the determination of reference values [38,39]. Furthermore, other analytic methods using mass spectrometry were not available for routine use in hospitals and outpatient facilities [40]. A conclusion on the basis of our study, the first one using the general cohort of patients prior to surgery, and table 2, five studies in CKD patients and one study in patients undergoing chemotherapy, can be that serum hepcidin levels may serve as a predictor for the response to iron therapy and correlates with serum ferritin.

To conclude, the current retrospective pilot study suggests that hepcidin might play an important role in the preoperative management of anemic patients. The presented study is a pilot study to screen for potential biomarkers to predict the responsiveness to IV iron. The difference in serum hepcidin levels of responders and non-responders is statistically noticeable and clinically relevant. The sample number is small. In the future, we aim to perform a prospective study with a higher sample size in order to analyze the properties of hepcidin as a biomarker (e.g. ROC analyses).
